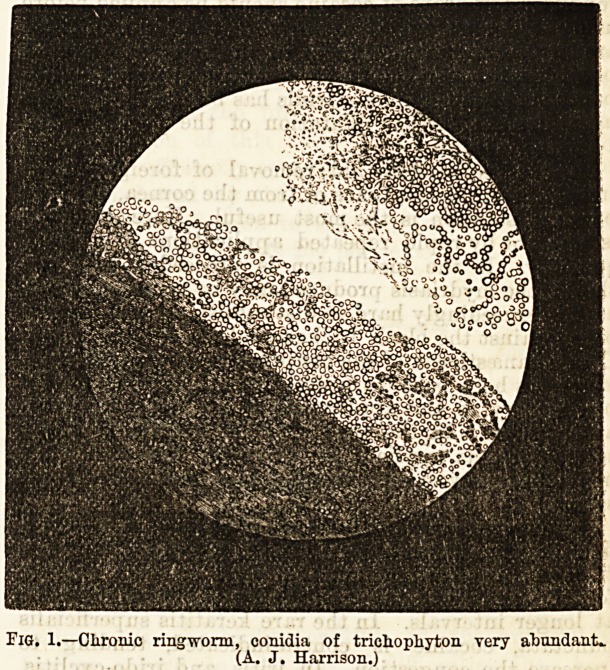# The Treatment of Ringworm at Bristol General Hospital and Elsewhere

**Published:** 1893-10-14

**Authors:** 


### THE TREATMENT OF RINGWORM AT BRISTOL GENERAL HOSPITAL, AND ELSEWHERE.

Two great difficulties in the treatment of ringworm render abortive all but the most careful and painstaking treatment. First, there are very few remedies which penetrate deeply enough to destroy the spores at the bottom of the hair follicles, and secondly, it is almost impossible to prevent dormant spores from the rest of the body and clothing from reinfecting the scalp. It is, however, remarkable that in drier climates, such as Germany and the United States, the disease is comparatively rare. We propose to summarise here the methods adopted by some of the chief dermatologists of the day, and then to describe one which has been found most useful in the Bristol General Hospital.

It appears that ringworm is not always due to the same species of trichophyton, many of the more intractable cases are the result of infection by a variety which differs in several respects from the common form, and whose powers of resistance are specially great. This may explain some of the discrepancies in the success obtained by different methods of treatment. Nothing but constant perseverance will effect a cure when the disease assumes an inveterate type, especially if opportunities of reinfection are present. Shoemaker in the drier climate of the United States is averse to shaving the head, and warns his pupils against even washing the hair. If possible the child must not live in damp houses, or in rooms where there is steam, or where the floors are often washed. He rubs in thoroughly, night and morning, a 50 per cent. solution of boro-glyceride. This will in mild cases suffice to effect a cure. The lotion should be applied to the entire scalp of every child in the house, as an aid to prevent infection of others and reinfection of the patient. For the spots where the disease appears most active, he prefers oleate of copper, with from four to nine parts of vaseline, and of this he rubs in a small quantity thoroughly night and morning. Occasionally he finds cases which answer better under a 5 per cent. oleate of mercury ointment, but in all he dresses the rest of the head with boro-glyceride. In the worst type of all he paints on croton oil over very small patches daily with a camel-hair brush, and poultices until a kerion is produced, to which the oleate of copper is again applied. Great care is needed not to treat more than a very small area at once in this way.

Radcliffe Crocker agrees in the necessity for the prevention of reinfection as of the utmost importance. He has the head washed three times a week with carbolised soft soap, and epilates the diseased hairs, but forbids brushing, which may loosen healthy ones and favour the extension of the disease. The patient must wear a cap lined with tissue paper frequently changed, and if possible should be isolated. Woollen curtains, scarves, and other clothes should be removed, as they furnish a resting-place for the spores, and the heads of the persons with whom the patient lives should be dressed daily with an alcoholic solution of borax, or a pomade containing ten grains to the ounce of thymol, for the same reason.

To the worst patches he applies an ointment of carbolic acid and sulphur ointment, followed by citrine ointment, well rubbed in. Any of the tried parasiticides in a vehicle of lanolin and olive oil may be used. A. carbolici, A. salicylici, aa. 3 grs., lanolini et vaselini aa. ℥ ss., ft. ung., or Coster's paste may be applied. The black crust which it causes must be removed, and soft soap must be applied to the spot afterwards.

Dr. Jamieson speaks highly of an ointment composed of precipitated sulphur ʒ 1, ammoniate of mercury 3 grs., thymol gr. x., vaseline ʒ 1, and simple ointment to an ounce. This is applied twice a day with friction. In many cases he uses an alcoholic solution of borax, made with one part of ether to four of spirit as a dressing two or three times daily, but recognising the inefficiency of all these plans in obstinate forms of the disease, he was led to the idea of developing sulphurous acid in the epidermal tissues themselves, so as to come nto contact with the spores in its nascent condition. With this end in view he washes the scalp with soft soap daily, so as to remove the grease entirely, and sponges it with dilute acetic acid twice in the twenty-four hours. While still damp he dabs it with a pledget of lint soaked in a solution of hyposulphite of soda (ʒ 1 to ℥ 1), to which a little glycerine has been added.

Before considering a similar plan, which has proved very efficacious in Dr. Harrison's clinique at Bristol, we may just advert to Eddowe's modification of Unna's method. During the first week he washes the scalp

Fig. 1.—Chronic ringworm, conidia of trichophyton very abundant. (A. J. Harrison.)

with soft soap two or three times, and applies daily an ointment formed of a drachm of sulphur in an ounce of vaseline. The hair is cut short and a cap worn. In subsequent weeks a chrysarobin ointment is rubbed daily into increasingly large patches, and the sulphur applied to the rest, but after four days, or sooner if irritation is produced, the sulphur ointment is entirely substituted. The skin should regain its normal colour before the chrysarobin is reapplied. The entire process is then repeated the next week. For the ointment the proportions are chrysarobin and icthyol aa gr. 25, and salicylic acid, gr. 10 to the ℥ 1.

## Figures and Tables

**Fig. 1. f1:**